# Between arteries, silent but possibly deadly: late presentation of a high-risk anomalous origin of a coronary artery

**DOI:** 10.1093/ehjcr/ytag797

**Published:** 2026-10-23

**Authors:** João Pedro Faria, Bárbara Rocha, Carla Marques Pires, Catarina Vieira

**Affiliations:** Cardiology Department, Hospital de Braga, São Victor, Rua das Sete Fontes, Braga 4710-243, Portugal; Cardiology Department, Hospital de Braga, São Victor, Rua das Sete Fontes, Braga 4710-243, Portugal; Cardiology Department, Hospital de Braga, São Victor, Rua das Sete Fontes, Braga 4710-243, Portugal; Cardiology Department, Hospital de Braga, São Victor, Rua das Sete Fontes, Braga 4710-243, Portugal

**Keywords:** Anomalous origin, Right coronary artery, Ischaemia, Congenital

## Summary

Anomalous origins of the coronary arteries are rare congenital conditions that may be associated with myocardial ischaemia and sudden cardiac death. We report the case of a 72-year-old man with atypical chest pain and inducible ischaemia on stress testing. Coronary computed tomography angiography (CCTA) revealed an anomalous origin of the right coronary artery from the left sinus of Valsalva with an acute-angle take-off and interarterial course, causing dynamic compression. This case highlights the diagnostic value of CCTA and the challenges of management in symptomatic older patients.

## Case description

A 72-year-old man was referred to a Cardiology consultation due to atypical chest pain and an exercise stress test suggestive of silent myocardial ischaemia. A transthoracic echocardiogram revealed preserved biventricular systolic function, with no regional wall motion abnormalities. A dobutamine stress echocardiogram demonstrated inducible ischaemia in the inferior wall, with chest discomfort and ST-segment depression in DII, DIII, aVF, V5, and V6. As the patient refused a coronary angiography, a coronary computed tomography angiography (CCTA) was performed, incidentally revealing an anomalous origin of the right coronary artery from the left Valsalva sinus, with a sharply angled take-off and an interarterial course between the aorta and pulmonary artery (*Figure 1*). There was visible narrowing of the proximal segment, consistent with dynamic external compression.

**Figure 1:**
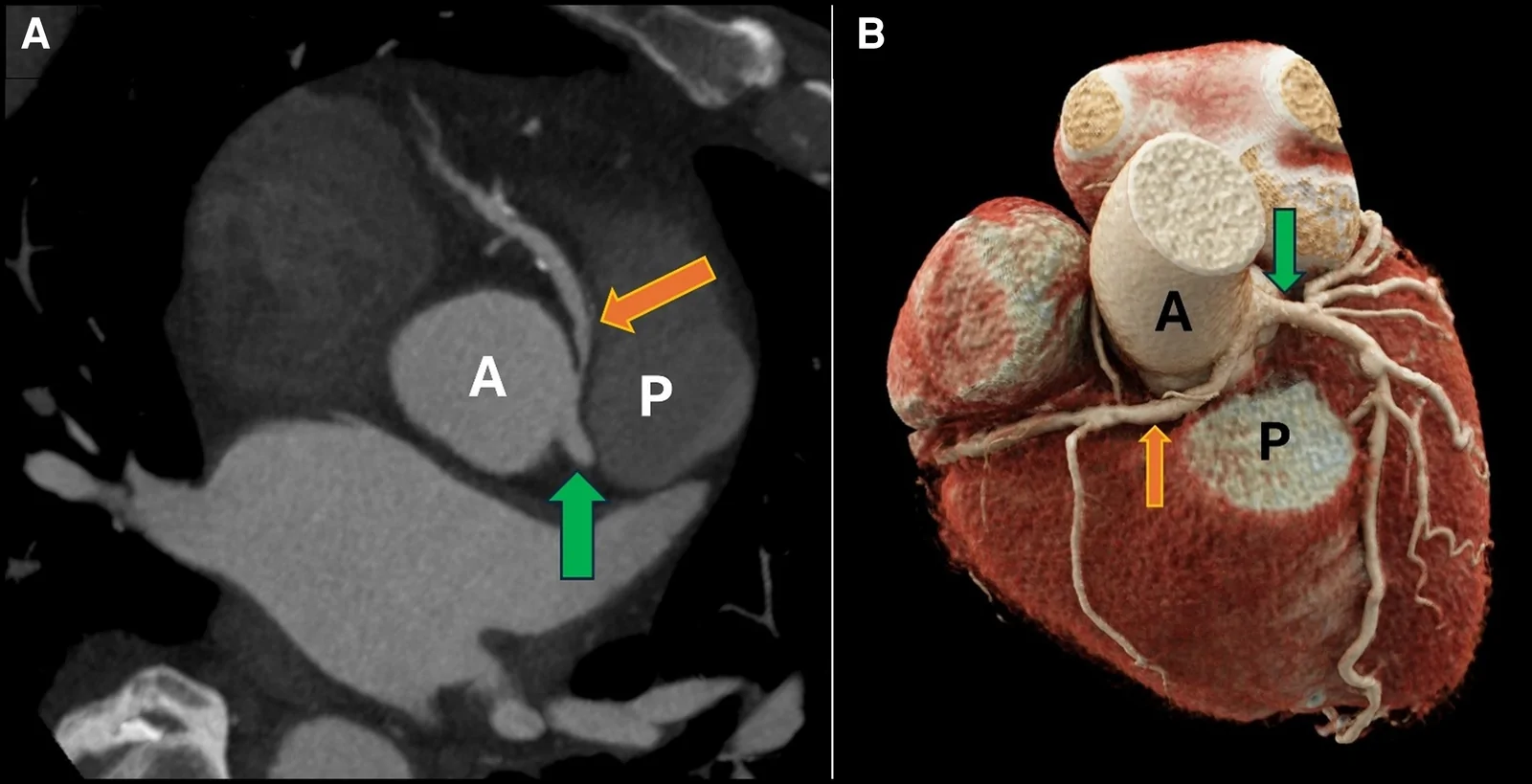
(*A*) Coronary computed tomography angiography in axial view, displaying the anomalous origin of the right coronary artery from the left Valsalva sinus, at a 29° angle. (*B*) 3D volume rendering technique of a coronary computed tomography angiography, displaying the anomalous origin of the right coronary artery from the left Valsalva sinus. A—aorta; P—pulmonary artery; orange arrow—right coronary artery; green arrow—left main coronary artery.

This variant is recognized as high risk for myocardial ischaemia and sudden cardiac death, particularly when the artery follows an interarterial course, with a slit-like orifice and acute-angle take-off.^1^ Current guidelines suggest that surgery should be considered even if the patient is asymptomatic.^2^ Therefore, the patient was proposed for surgical correction, but opted for conservative management, and was started on aspirin, rosuvastatin, and nebivolol. He remained asymptomatic, with no cardiac events after 3 years of follow-up.

This case illustrates a rare but potentially life-threatening coronary anomaly. CCTA plays a key role in diagnosis and surgical planning,^3^ as coronary anomalies with an interarterial course and acute angle of origin carry important prognostic implications. However, it is questionable whether surgery in newly symptomatic older patients provides any survival benefit. Therefore, management should be individualized, balancing anatomical risk, clinical presentation, and patient preference. This case underscores the importance of vigilance and long-term follow-up, even in asymptomatic individuals, as the clinical course is still incompletely understood.

## Data Availability

All data relevant to the study are included in the article. **Ethics approval:** This is an image report. The Research Ethics Committee of Hospital de Braga has confirmed that no ethical approval is required.
